# Systemic Outcomes in Adults Undergoing Emergent Repair of Orbital Blowout Fractures

**DOI:** 10.1007/s12070-024-04681-0

**Published:** 2024-04-20

**Authors:** Owais M. Aftab, Avneet Randhawa, Karandeep S. Randhawa, Imran M. Khawaja, Keshav Kumar, Paul D. Langer, Jean Anderson Eloy, Christina H. Fang

**Affiliations:** 1 Department of Otolaryngology- Head and Neck Surgery, Rutgers New Jersey Medical School, Newark, NJ USA; 2 Department of Ophthalmology and Visual Science, Rutgers New Jersey Medical School, Newark, NJ USA; 3 Department of Otorhinolaryngology - Head and Neck Surgery, Albert Einstein College of Medicine, Bronx, NY USA; 4 Center for Skull Base and Pituitary Surgery, Neurological Institute of New Jersey, Rutgers New Jersey Medical School, Newark, NJ USA; 5 Department of Neurological Surgery, Rutgers New Jersey Medical School, Newark, NJ USA; 6 Department of Otolaryngology and Facial Plastic Surgery, Saint Barnabas Medical Center – RWJ Barnabas Health, Livingston, NJ USA; 7 Department of Otorhinolaryngology – Head and Neck Surgery, Montefiore Medical Center, The University Hospital of Albert Einstein College of Medicine, 3400 Bainbridge Avenue Medical Arts Pavilion, 3rd Floor, Bronx, NY 10467 USA

**Keywords:** Orbital blowout fractures, Emergent repair, Systemic outcomes, Postoperative outcomes, NSQIP

## Abstract

**Purpose:**

To analyze the association between emergent surgery status and systemic adverse outcomes in patients undergoing open orbital floor blowout fracture repair.

**Methods:**

This retrospective cohort analysis utilized the 2005–2018 National Surgery Quality Improvement Program (NSQIP) database. Current Procedural Terminology (CPT) codes were used to identify cases with open treatment of orbital floor blowout fractures (21385, 21386, 21387, 21390, 21395). Demographics, comorbidities, and complication incidences were compared between patients undergoing emergent surgery and those undergoing non-emergent orbital blowout fracture repair using chi-square analyses. The independent effect of preoperative emergent status on adverse outcomes was analyzed using binary logistic regression.

**Results:**

1,146 (96.0%) non-emergent and 48 (4.0%) emergent orbital blowout fracture repairs were identified from 2005 to 2018. Chi-square analysis indicated patients undergoing emergent repairs had higher incidences of preoperative wound infection (8.3% vs. 2.3%; *p* = 0.029) and systemic sepsis (8.3% vs. 0.6%; *p* = 0.001). The emergent cohort had a higher proportion of patients with Hispanic ethnicity (*p* = 0.011). Unadjusted chi-square analysis indicated the emergent cohort had a higher incidence of prolonged length of stay (50.1% vs. 10.1%; *p* < 0.001). After adjusting for confounders, logistic regression analysis indicated emergent status was an independent risk factor for prolonged length of stay (OR 13.05; 95% CI 5.26–32.37; *p* < 0.001).

**Conclusion:**

Emergent surgery status is an important factor associated with increased odds of prolonged length of stay in patients undergoing open orbital blowout fracture repair.

**Supplementary Information:**

The online version contains supplementary material available at 10.1007/s12070-024-04681-0.

## Précis

This national database analysis evaluated over 1000 orbital blowout fractures cases and ascertained that emergent status of orbital blowout fracture repair outcomes is generally not associated with systemic complications in adults.

**Supplementary Information:**

The online version contains supplementary material available at 10.1007/s12070-024-04681-0.

## Introduction

Facial fractures are common among patients with head and neck trauma with a global incidence of over 7.5 million cases in 2017 [1]. In the United States, facial fractures composed approximately 5% of 5 million head and neck trauma cases presenting to the emergency department in 2011 [2]. Orbital fractures in particular, are among the most common fractures secondary to facial trauma [1, 3, 4]. Orbital fractures most commonly involve areas where the bones of the orbit are the weakest (for example, just medial to the infraorbital neurovascular bundle). The most common type of orbital fracture is a blowout fracture, which is defined as a fracture of the orbital floor where the inferior orbital rim is intact [5]; this is contrast to, for example, the less common blow-in fracture, where the orbital rim is compromised with a resultant decrease in orbital volume [6].

Generally, the management of orbital blowout fractures in stable patients without any other clinically concerning injuries is not emergent and can be performed in an outpatient setting [7]. Indications for surgery include an orbital floor defect greater than 1 cm^2^, early enophthalmos, significant hypoglobus, or persistent diplopia, although, surgery is typically delayed by a week or more to allow swelling and ecchymosis to subside [8]. However, immediate clinical evaluation is key, because certain sequalae such as extraocular muscle entrapment or traumatic optic neuropathy are emergent issues that may require more urgent intervention. Specifically, patients with orbital floor trap-door blowout fractures treated within 8 days were found to have better surgical outcomes [9].

To date, no studies have specifically evaluated the impact of emergent status of orbital blowout fractures as a factor on postoperative adverse outcomes using a national database. We hope to fill this gap in the literature through this analysis.

## Materials and Methods

### Database

This retrospective cohort analysis utilized the 2005–2018 National Surgery Quality Improvement Program (NSQIP). Data from NSQIP is obtained from over 680 hospitals across the United States and includes over 250 peri-operative variables. Data captured includes demographic variables, preoperative comorbidities, and postoperative complications up to 30 days after surgery, including 30-day morbidity and mortality, on over 1 million cases per year [10]. Data from NSQIP can play a key role in identifying areas for improvement in surgical outcomes or confirming the safety of surgical procedures [11]. Because the data in NSQIP is de-identified, this study was deemed as exempt by the Rutgers New Jersey Medical School Institutional Review Board. As such, this study was HIPAA compliant and adhered to the tenets of the Declaration of Helsinki.

### Study Population

Current Procedural Terminology (CPT) codes were utilized in this study to identify cases with a primary procedure of orbital floor blowout fracture repair. CPT codes utilized included a transantral approach (21385), periorbital approach (21386), combined approach (21387), periorbital approach with alloplastic or other implant (21390), and periorbital approach with bone graft (21395) in accordance with prior NSQIP analyses evaluating orbital blowout fractures [12]. Note that we only included cases with a primary procedure of orbital blowout fracture repair to limit any confounding impact other primary procedures may have on patient outcomes [11]. Cases with missing data were also excluded from this analysis.

Cohorts were stratified by emergent status. Emergent was defined according to the NSQIP criteria of an emergent case, which aligns with prior literature evaluating outcomes of emergent surgeries [13]. NSQIP labels a case as emergent when the primary surgeon determines a case to be emergent. Specifically for orbital fracture repair, NSQIP labeling of emergent status include cases with operation in the same hospital stay that also have a clinical identification of indications for emergent operative repair such as extraocular muscle entrapment, orbital compartment syndrome, and/or other severe entrapment.

### Study Variables

Preoperative variables utilized in this study included demographic variables and other confounding medical conditions. Demographic variables evaluated included gender, age (18–34, 35–49, 50–65, 65 + years), race (White, Black, Asian, Hawaiian/Pacific, Native American, Unknown), and preoperative comorbidities included obesity, diabetes, smoking, dyspnea, poor functional status (defined as a partially or totally dependent health status prior to surgery), ventilator dependence, chronic obstructive pulmonary disease, ascites, congestive heart failure, hypertension, renal failure, dialysis, disseminated cancer, open wound(s), steroid use, weight loss, bleeding disorders, preoperative blood transfusion, and preoperative systemic sepsis. Postoperative outcomes evaluated in this study included medical complications and surgical complications. Medical complications included progressive renal insufficiency, stroke/cardiovascular accident, cardiac arrest, myocardial infarction, urinary tract infection, septic shock, sepsis, pneumonia, pulmonary embolism, acute renal failure, unplanned intubation, and deep venous thrombosis. Surgical complications included intraoperative or postoperative transfusion, superficial incisional surgical site infection (SSI), deep incisional SSI, organ space SSI, and wound disruption. Complications including any surgical complication, any medical complication, mortality, prolonged length of hospital stay (LOS) (defined as an LOS greater than the 90th percentile of selected cases), prolonged operation time (defined as an operation time greater than the 90th percentile of selected cases), unplanned reoperation, and unplanned readmission were also included.

### Statistical Analysis

Demographics and preoperative comorbidities were first evaluated in patients undergoing orbital blowout fracture repair by emergent or non-emergent status using Pearson chi-square analysis and Mann-Whitney U-tests as appropriate. Similarly, unadjusted chi-square analysis was used to evaluate associations between emergent status and adverse postoperative outcomes. Preoperative demographics and comorbidities found to significantly vary between cohorts were used as confounding variables in a multivariate, binary logistic regression model evaluating the influence of emergent patient status on adverse outcomes. In accordance with past literature, a minimum of 10 events per variable (EPV) for logistic regression was confirmed prior to conducting binary logistic regression to ensure adequate power [14]. An alpha level of *p* < 0.05 was used. All statistical analysis was carried out in SPSS Statistics Version 25.0 (IBM Corporation, Armonk, NY).

## Results

1,146 (96.0%) non-emergent and 48 (4.0%) emergent orbital blowout fracture repairs were identified from 2005 to 2018. 23.4% (n = 268) of non-emergent cases were performed as an inpatient, as compared to 54.2% (n = 26) of emergent cases (*p* < 0.001). Furthermore, chi-square analysis indicated that gender did not significantly vary in the cohorts (*p* = 0.654) (Table 1), while age significantly varied among cohorts (*p* = 0.019). Patients aged 18–35 years made up 41.4% of the non-emergent and 50.0% of the emergent cohorts, while patients aged 65 + made up 9.9% as well as 20.8% of the non-emergent and emergent cohorts, respectively. Race also significantly varied among cohorts (*p* < 0.001), as unknown cases made up 38.6% of the emergent cohort and 13.9% of the non-emergent cohort. Notably, the proportion of Hispanic ethnicity also varied in cohorts, as Hispanic cases made up 25.0% of emergent patients as compared to 8.8% of non-emergent patients (*p* = 0.011).

**Table 1:** Demographics and comorbidities of patients undergoing orbital blowout fracture repair according to emergent status

	Non-Emergent	Emergent	*p*-value
Gender			0.653
Female	39.0%	35.4%	
Male	61.0%	64.6%	
Age cohorts, years			**0.019**
18–34	41.4%	50.0%	
35–49	24.2%	14.6%	
50–65	24.6%	14.6%	
65+	9.9%	20.8%	
Race			**< 0.001**
White	60.0%	52.3%	
Black	20.6%	6.8%	
Asian	3.3%	0.0%	
Hawaiian/Pacific	0.8%	0.0%	
Native American	1.5%	2.3%	
Unknown	13.9%	38.6%	
Obese	31.6%	33.3%	0.870
Diabetes mellitus	3.4%	2.1%	1.000
Smoker	36.7%	39.6%	0.760
Dyspnea	2.4%	0.0%	0.623
Poor functional status	0.9%	0.0%	1.000
Ventilator dependence	N/A	N/A	N/A
Chronic obstructive pulmonary disease	2.6%	2.1%	1.000
Ascites	N/A	N/A	N/A
Congestive heart failure	0.3%	0.0%	1.000
Hypertension	25.4%	22.9%	0.865
Renal failure	0.1%	0.0%	1.000
Dialysis	0.3%	0.0%	1.000
Disseminated cancer	0.1%	0.0%	1.000
Open wound	2.3%	8.3%	**0.029**
Steroid use	1.6%	0.0%	1.000
Weight loss	0.1%	2.1%	0.079
Bleeding disorder	0.8%	2.1%	0.338
Preoperative blood transfusion	0.2%	2.1%	0.116
Systemic sepsis	0.6%	8.3%	**0.001**

The non-emergent and emergent cohorts were then evaluated using chi-square analysis to identify any comorbidities that significantly varied between cohorts. The cohorts generally had similar incidences of preoperative comorbidities, but the emergent cohort had a significantly higher incidence of open wound(s) (8.3% vs. 2.3%; *p* = 0.029) and systemic sepsis (8.3% vs. 0.6%; *p* = 0.001). However, other conditions such as obesity, diabetes, and/or smoking status, among other variables, did not vary significantly among cohorts (Table 1).

**Fig. 1:**
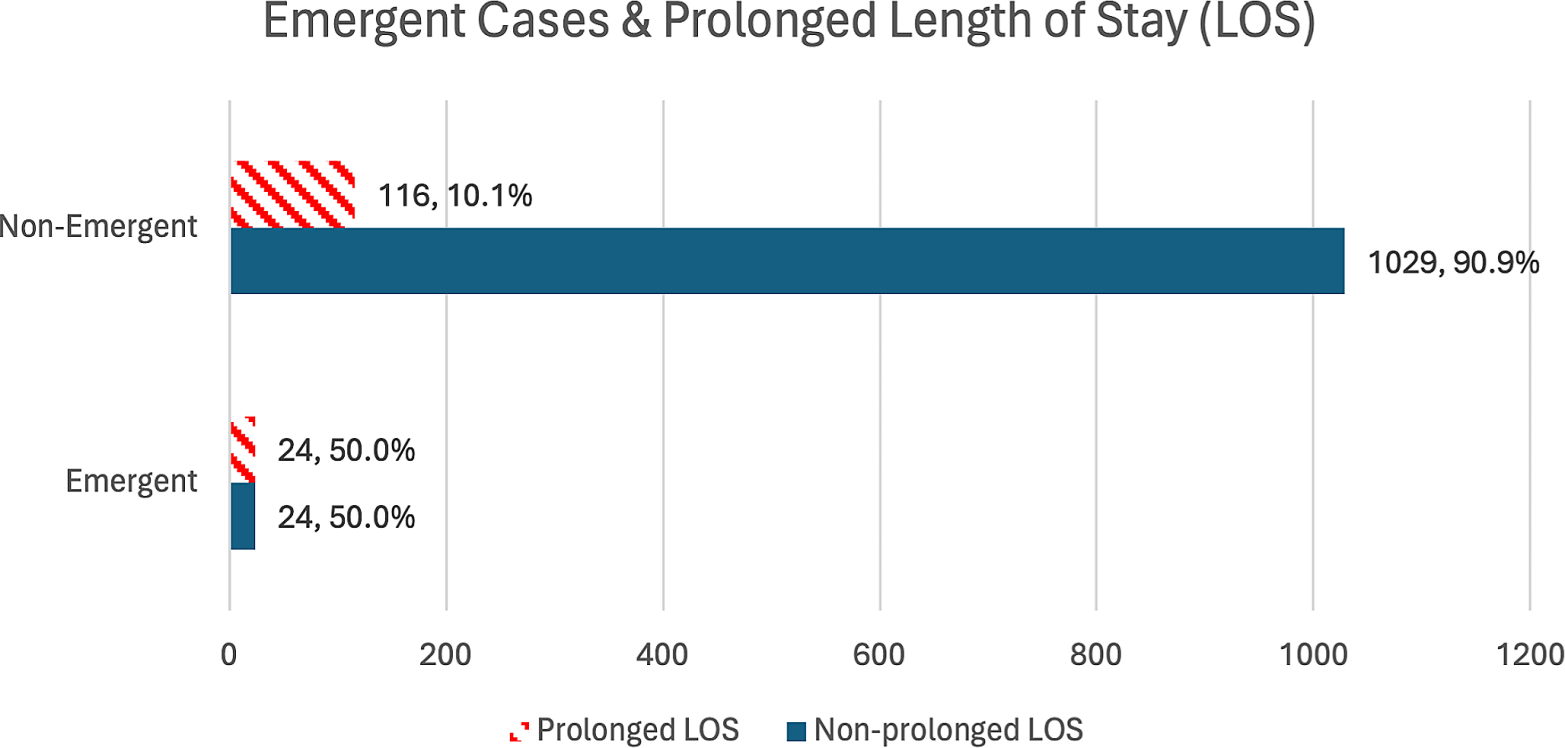
Emergent cases & prolonged length of stay (LOS)

On unadjusted chi-square analysis, it was found that patients undergoing emergent orbital blowout fracture repair did not significantly vary from the non-emergent cohort (Table 2) in postoperative surgical outcomes such as any surgical, medical, or any complications. Cohorts similarly did not differ in mortality or in the proportion of prolonged operation time, unplanned reoperation, and unplanned readmission. However, 50.0% of the emergent cohort had prolonged LOS, as compared to 10.1% of the non-emergent cohort (*p* < 0.001) (Fig. 1). While we found that the mean overall LOS was 0.94 days ± 3.68 days (median of 0.00 days and interquartile range of (0.00, 1.00) days), on unadjusted Mann-Whitney U-tests, the emergent cohort had a significantly longer median LOS (0.00 days vs. 1.50 days; U = 37,684.500; *p* < 0.001).

**Table 2:** Chi-square analysis of complication incidences in patients undergoing orbital blowout fracture repair according to emergent surgery status

	Non-Emergent	Emergent	*p*-value
Any surgical complication	0.6%	0.0%	1.000
Any medical complication	0.6%	0.0%	1.000
Any complication	1.1%	0.0%	1.000
Death	0.3%	2.1%	1.000
Prolonged length of stay	10.1%	50.1%	**< 0.001**
Prolonged operation time	10.0%	10.4%	0.809
Unplanned reoperation	2.4%	4.2%	0.327
Unplanned readmission	3.0%	3.4%	0.589
Length of Stay (Median, IQR)	0.0 (0.0, 1.0) days	1.5 (0.0, 3.0)	**< 0.001**
Operative Time (Median, IQR)	72.0 (48.0, 106.0) min	77.5 (51.3, 98.5) min	0.883

SSI – surgical site infection; IQR, interquartile range

*No cases were reported in NSQIP for these complications

After adjusting for all demographic variables and comorbidities that significantly varied among the non-emergent and emergent cohorts on multivariate, binary logistic regression, we found that the emergent cohort had a significantly higher odds of a prolonged LOS (OR 12.696; 95% CI 5.083–31.710; *p* < 0.001). No other significant differences in adverse outcomes were found between the two cohorts, including in any surgical complications (OR 0.000; 95% CI 0.000 – NA; *p* = 0.998), any medical complications (OR 0.000; 95% CI 0.000 – NA; *p* = 0.998), any complications (OR 0.000; 95% CI 0.000 – NA; *p* = 0.998), death (OR 12.970; 95% CI 0.888–180.384; *p* = 0.061); prolonged operative time (OR 2.444; 95% CI 5.083–31.710; *p* < 0.001), unplanned reoperation (OR 3.749; 95% CI 0.628–22.401; *p* = 0.147), or unplanned readmission (OR 4.996; 0.555–44.976; *p* = 0.151) (Table 3).

**Table 3:** Binary logistic regression analysis of adverse outcomes in patients undergoing emergent vs. non-emergent orbital blowout fracture repair

Outcome	Odds Ratio	95% Confidence Interval	*p*-value
Any surgical complication	0.000	0.000 – NA*	0.998
Any medical complication	0.000	0.000 – NA*	0.998
Any complication	0.000	0.000 – NA*	0.998
Death	12.970	0.888–180.384	0.061
Prolonged length of stay	12.696	5.083–31.710	**< 0.001**
Prolonged operation time	2.444	0.781–7.646	0.125
Unplanned reoperation	3.749	0.628–22.401	0.147
Unplanned readmission	4.996	0.555–44.976	0.151

SSI – surgical site infection

*Odds ratios could not be reported due to limited number of cases

## Discussion

In this analysis, we evaluated the impact of emergent status on outcomes of orbital blowout fracture repair. We found that the majority of orbital blowout fracture repairs were coded as non-emergent in NSQIP, which is in accordance with previous literature indicating that the majority of orbital blowout fractures do not require emergent intervention [7, 15–17]. Furthermore, we focused on orbital blowout fractures in adults this analysis, which completely excludes pediatric “trapdoor” fractures that can present more urgently [8]. However, it is important to note that some surgeons prefer to manage orbital blowout fracture patients in the inpatient setting out of concern for prompt management of intraorbital hemorrhage [18], although the cost-benefit analysis from a hospital standpoint is not clear due to an apparently low risk of intraorbital hemorrhage and other complications in orbital blowout fracture repair [15].

We found that both emergent and non-emergent fractures were present in a higher proportion in males and in the 18-35-year-old demographic. This is also aligned with past literature as a past NSQIP analysis of facial fractures, inclusive of orbital blowout fractures, found that fractures occur more frequently in the male demographic and in younger patients rather than older patients [12]. Interestingly, we found that the emergent cohort significantly differed from the non-emergent cohort, in that the emergent cohort had a higher proportion of individuals aged 65+. This may indicate that although orbital blowout fractures in general are more common in younger patients, orbital blowout fractures may be more likely to be managed as emergent conditions in the elderly. This could be due to a myriad of factors, as the elderly are known to have a greater risk of complications following fractures in general [19]. More specifically, while extraocular muscle entrapment is the most common indication for emergent repair of orbital floor fracture repair, especially in the pediatric population, in adults, this complication tends to be more uncommon. We suspect that an age-based difference may partly be due to an increased frequency of orbital compartment syndrome, extraocular muscle entrapment, and/or severe entrapment in the elderly cohort. This assertion is partly supported by past literature demonstrating a mean patient age of 67.41 of orbital compartment syndrome patients [20]. It is additionally possible that age may play a role in the severity of orbital fracture secondary to injury due to a well-known association between advanced age and osteoporosis [21]. Such concerns inform a more conservative approach in the elderly, which could also play a role in an increased likelihood of emergent classification of emergent fractures in this demographic.

We found that emergent and non-emergent cohorts generally did not vary in comorbidities. This aligns with prior literature which evaluating risk factors for orbital fracture as it was found that clinical features such as blunt trauma injury, inability to count, roof fracture, diplopia, and/or conjunctival hemorrhage, in addition to other clinical and radiographic findings, suggested a serious risk of ocular injury, though most patients presenting to the emergency room did not require urgent consultation [17, 22, 23].

Furthermore, we found that emergent and non-emergent patients did not significantly differ in any postoperative surgical complications other than LOS. While slightly unexpected, this concurs with other literature which suggests that emergent patients with orbital fracture have a low incidence of postoperative complications and could be candidates for surgical repair on an outpatient basis [15]. Additionally, other past analyses of orbital fracture repair have considered the possibility of managing orbital floor fractures on an outpatient basis, as it has been found that time to repair of fracture does not impact postoperative outcomes [24]. It is important to note, however, that significant variability in orbital floor fracture management exists as some recommend management on an emergent basis while others recommend waiting for at least one week [8, 18].

Despite variability in management, we found that all orbital blowout fracture patients had an average LOS of 0.94 days, which is similar to values previously reported in literature. For instance, in a single-center evaluation of the safety of outpatient isolated orbital fracture repair, average hospital LOS of orbital floor fracture was found to be 0.85 days [15]. Patients can expect to resume normal activity approximately three weeks after fracture repair [25].

We also found that, despite not having an increased incidence of adverse postoperative surgical outcomes, emergent patients did have a prolonged LOS. This is salient because prolonged lengths of stay frequently translate to increased costs [26]. Emergent orbital blowout fracture patients with a severe, initial presentation may be a focus of increased medical attention [15], and conservative management of orbital blowout fractures in this cohort may explain our finding for a difference in LOS despite no differences in morbidity and/or mortality. However, this is an expected finding as indications for emergent orbital fracture repair naturally select for more complicated cases involving extraocular muscle entrapment, orbital compartment syndrome, or other severe features.

Our study contains several limitations, several of which are inherent to retrospective, national database analyses. For example, we were unable to identify the free text beyond a surgeon’s labeling of an orbital fracture case as being an emergent case due to NSQIP’s codification of a simple yes/no status behind emergent cases. Further, we were unable to consider extraocular muscle entrapment, visual acuity, or other outcomes specifically relevant to orbital blowout fracture repair as postoperative endpoints in this study because these conditions are not coded for in NSQIP. Despite this, evaluations of NSQIP can yield great insight due to its representation of data from a national level. While it may not be feasible to fully address the aforementioned limitations, we did try to mitigate this by specifically including cases with a primary procedure of orbital blowout fracture repair, rather than generalizing inclusion criteria to avoid excessive heterogeneity/variation of our sample in this study, though in doing so, we limited the sample size of our study.

## Conclusion

In this retrospective, national analysis, we evaluated postoperative complications associated with emergent status in orbital blowout fracture repair. We found that cohorts differed in prolonged LOS but not in morbidity or mortality on both unadjusted univariate and adjusted multivariate analyses. We found differences between emergent and non-emergent cohorts may be less pronounced than in other surgeries and that orbital blowout fracture repair generally does not involve systemic complications.

### Electronic Supplementary Material

Below is the link to the electronic supplementary material.

Supplementary Material 1
