# Supplementary material for: Systemic Outcomes in Adults Undergoing Emergent Repair of Orbital Blowout Fractures
Source: Indian J Otolaryngol Head Neck Surg. 2024 Apr 20;76(4):3323–9. doi: 10.1007/s12070-024-04681-0 (PMC11306889; doi:10.1007/s12070-024-04681-0)
Supplement: Supplementary file 1 — Supplementary Material 1 [file 12070_2024_4681_MOESM1_ESM.docx]

**Observations:**

1. **Keywords:** For uniformity of the journal style and accessibility on search engines, please use MeSH keywords. Following MeSH Keywords would have been ideal: **Orbital Blowout Fractures, Emergent Repair, Systemic Outcomes, Postoperative Outcomes, NSQIP**.
   1. We appreciate the Editor’s comments and insight on this submission. We are happy to revise the keywords. See Lines 27-28
2. **Statistical analysis:** The described statistical approach is comprehensive for the stated objectives, but there are a few considerations and potential additions that could enhance the robustness and clarity of the analysis.
   1. We appreciate the insight from the Reviewer.
3. **Effect Size and Confidence Intervals:** Alongside p-values, reporting effect sizes and confidence intervals provides a more complete picture of the results. This is particularly important in logistic regression to understand the magnitude and precision of the effects.
   1. We appreciate the feedback from the reviewer. We reworked the Results section so that effect sizes are included for all mentions of binary logistic regression. See lines 128-146.
4. **Power Analysis:** Mention of a power analysis to ensure the study is adequately powered to detect meaningful effects might be relevant, especially in studies with smaller sample sizes.
   1. In accordance with past literature, a minimum of 10 events per variable (EPV) for logistic regression was confirmed prior to conducting binary logistic regression to ensure adequate power. See Lines 108-111. We also noted our study’s limited sample size as a limitation. See Lines 221-222.
5. **Subgroup analyses:** The primary outcome of this study is Length of Stay (LOS) between emergent and non-emergent cases. Therefore, a subgroup analysis involving logistic regression to examine the relationship between indications for emergent surgery, such as the extent of injury and other factors, and LOS might have provided additional insights.
   1. We appreciate the reviewer’s comment on a subgroup analysis involving logistic regression to evaluate the relationship between indications for emergent surgery (namely, extent of injury and other factors) to provide other insight. Unfortunately, the NSQIP database is limited in the information it provides on emergency surgeries, encoding them simply as a binary variable rather than as a text field where it is possible to parse out the exact indication for emergent surgery. This limitation has been added to the limitations section. See lines 211-220.
6. **Data Visualization:** While not a part of statistical testing per se, including data visualization (like graphs or charts) could have helped in effectively communicating key findings.
   1. See Figure 1, a visual depiction to communicate key findings. Note that we would be happy to remove this if the editors feel it is of limited value.
7. **Desired but not a must:** Only generic terminologies such as "some surgeons" or "other literature" may be more appropriate than citing specific names to maintain objectivity and neutrality, ensure universality and relevance, and to avoid bias as well as for ethical considerations. However, if a specific method or theory developed by a particular individual or group is discussed, then it might be appropriate and necessary to cite them by name, especially if their work is directly relevant to the study.
   1. We appreciate this insight from the reviewer. To maintain objectivity and neutrality, we removed references to specific names/groups, and instead used generic terminology.
